# Rotator Interval Lesion and Damaged Subscapularis Tendon Repair in a High School Baseball Player

**DOI:** 10.1155/2015/890721

**Published:** 2015-11-05

**Authors:** Tomoyuki Muto, Hiroki Ninomiya, Hiroaki Inui, Masahiko Komai, Katsuya Nobuhara

**Affiliations:** Department of Orthopaedic Surgery, Nobuhara Hospital and Institute of Biomechanics, 720 Haze, Issai-cho, Tatsuno-shi, Hyogo-ken 679-4017, Japan

## Abstract

In 2013, a 16-year-old baseball pitcher visited Nobuhara Hospital complaining of shoulder pain and limited range of motion in his throwing shoulder. High signal intensity in the rotator interval (RI) area (ball sign), injured subscapularis tendon, and damage to both the superior and middle glenohumeral ligaments were identified using magnetic resonance imaging (MRI). Repair of the RI lesion and partially damaged subscapularis tendon was performed in this pitcher. During surgery, an opened RI and dropping of the subscapularis tendon were observed. The RI was closed in a 90° externally rotated and abducted position. To reconfirm the exact repaired state of the patient, arthroscopic examination was performed from behind. However, suture points were not visible in the >30° externally rotated position, which indicates that the RI could not be correctly repaired with the arthroscopic procedure. One year after surgery, the patient obtained full function of the shoulder and returned to play at a national convention. Surgical repair of the RI lesion should be performed in exactly the correct position of the upper extremity.

## 1. Introduction

Rotator interval (RI), as described in the book written by Post [1], is composed of capsular tissue spanning the interval between the superior aspect of the subscapularis (SSC) and anterior border of supraspinatus (SSP). The RI is formed by a thin, elastic, membranous tissue. The coracohumeral ligaments and capsule help strengthen the RI tissue. Nobuhara and Ikeda proposed that the RI lesion was a clinical disease entity that could often resemble a rotator cuff tear or recurring traumatic subluxation of the shoulder [2]. A RI lesion is extremely common among young throwing athletes [3]. The RI resists inferior translation of the humeral head in the adducted shoulder and posterior translation of the humeral head in the flexed or abducted and externally rotated shoulder and limits external rotation [4, 5]. Instability of the glenohumeral joint occurs inferiorly and posteriorly after section of the RI capsule. Imbrication of the RI capsule increased the resistance to inferior and posterior translation [5]. Various operation techniques for arthroscopic closure or open RI repair have been reported; however, a complication of these techniques is loss of external rotation. Here, we report a case of open RI repair in a 90° externally rotated and abducted position for a baseball pitcher leading to good clinical results without postoperative loss of external rotation.

## 2. Case Presentation

The subject was a 16-year-old (183 cm, 75 kg) male, amateur high school baseball pitcher, with right shoulder pain and decreasing range of motion in the right shoulder. He started to play baseball as an infielder or pitcher at the age of 10. His pitching style was overhand throwing and his maximum ball speed was 130 km/h. He had a 4-month history of loss of ball control with moderate pain over the anterior and posterior aspects of the shoulder at maximum external rotation. He had previously attended a local hospital for physiotherapy and received conventional exercises for strengthening his scapular muscles. However, he abandoned the exercise program a few months later because his ball control was not restored and his pain was not improved. He was admitted to our hospital in 2013.

**Figure 1:**
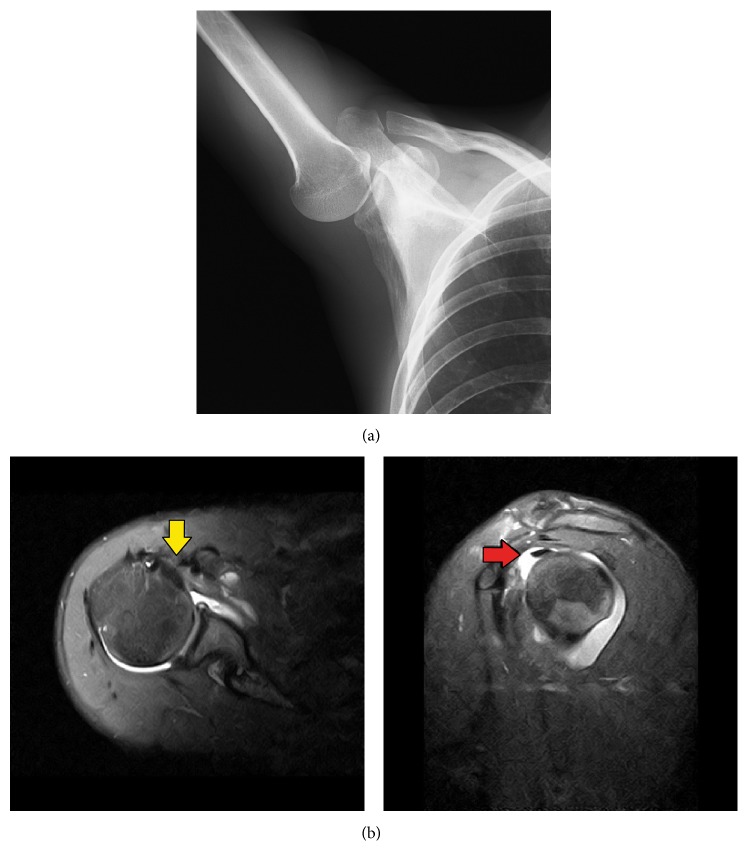
(a) X-ray image of the upper arm in the elevated position showing humeral head slipping. (b) MRI images of the superior aspect of the subscapularis tendon and muscle injury (yellow arrow) and rotator interval lesion (red arrow) (fat suppressed imaging).

The patient had the following 4 chief complaints: (1) severe deep anterior shoulder pain with maximum external rotation of the shoulder, (2) loss of ball control when pitching with deep anterior shoulder pain, (3) insufficient elevation of his elbow, (4) feeling that his shoulder was unstable from the cocking phase to follow-through. Posteroinferior instability (slipping) was observed in an X-ray of the upper arm in an elevated position (Figure 1(a)). According to the magnetic resonance imaging (MRI) scan, an SSC tendon and muscle injury and RI, superior glenohumeral ligament (SGHL), and middle glenohumeral ligament (MGHL) lesions were observed (Figure 1(b)). Based on these findings, the patient was diagnosed with RI lesion and SSC injury, and open RI repair was suggested.

**Figure 2:**
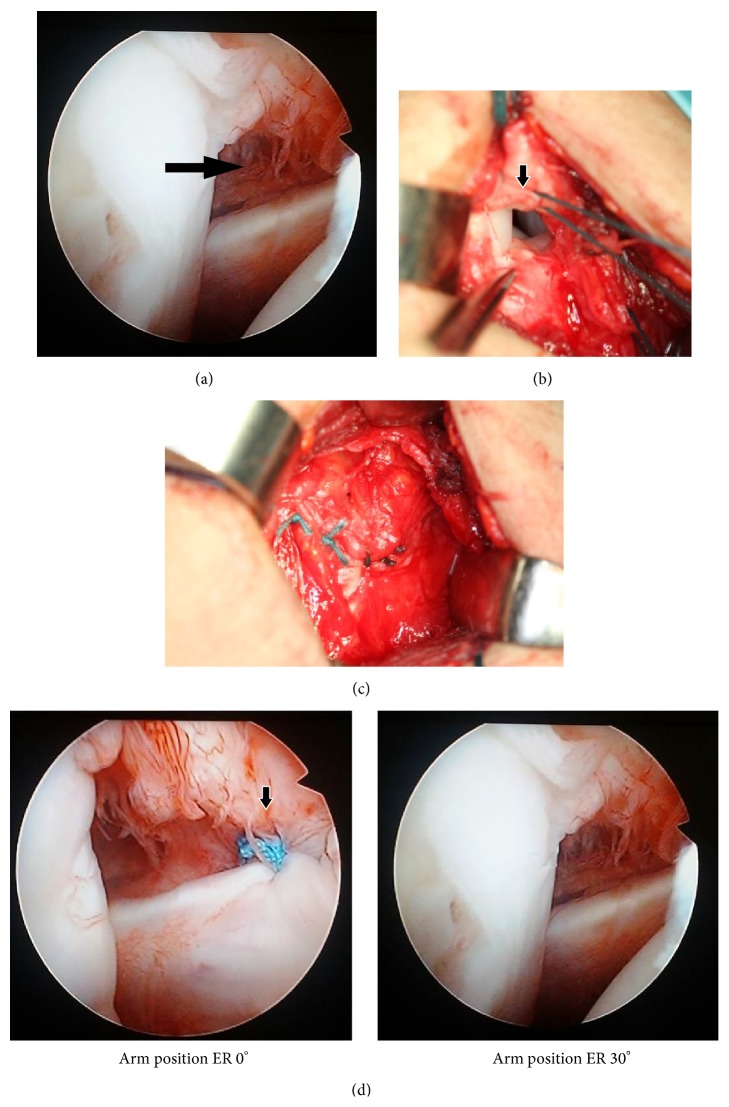
(a) Image of the rotator interval opened (black arrow), dropping of the superior aspect of the subscapularis, and the disappearance of the superior and middle glenohumeral ligament lesions obtained using arthroscopy. (b) An image showing the dell and abnormal slackness of the rotator interval (black arrow). (c) The coracohumeral ligament was pulled over repaired rotator interval and sutured in place. (d) Repaired rotator interval closure was observed in the 0° externally rotated (ER) position from behind (black arrow). It was not visible in the 30° ER position.

The patient was placed in the beach-chair position under general anesthesia. Firstly, standard diagnostic arthroscopy was performed through a posterior portal. Opening of the RI, dropping of the SSC, and disappearance of the SGHL and MGHL were observed using arthroscopy (Figure 2(a)). An anterolateral incision of 6 cm was performed one finger breadth lateral to the coracoid process. The deltoid muscle fibers were separated and the subacromial bursa was incised, but not resected. Adhesions of the subacromial bursa and a collapse and abnormal slackness of the RI were observed (Figure 2(b)). The RI was loose and could easily be grasped with forceps. The edges of the SSP and adjacent SSC were roughed and closed with two interrupted nonabsorbable sutures, while the shoulder was held in a 90° externally rotated and abducted position. The coracohumeral ligament was then pulled over the repaired RI and sutured in place (Figure 2(c)). The bursa, deltoid fascia, and skin were repaired with sutures. Afterwards, the RI closure point was rechecked using arthroscopy (Figure 2(d)).

The patient wore a sling for 3 weeks. Pain-free passive range of motion exercise and sling exercise were initiated at 2 weeks after surgery. Active range of motion exercise was permitted at 3 weeks, and a muscle-strengthening program was initiated 8 weeks after surgery. One year after the operation, the patient obtained full function of the shoulder, returned to full activity, and participated in a baseball national convention.

## 3. Discussion

The rotator interval is directly affected by changes in shoulder position. RI injury can occur when an athlete quickly returns to an internally rotated position from an excessively externally rotated position (i.e., pitching and spiking during baseball and volley ball, resp.) [6]. Various techniques for open RI repair or arthroscopic RI closure have been reported [2, 7, 8]. However, both open RI repair and arthroscopic RI closure have shown great loss in external rotation, especially in the neutral position [9]. In a cadaveric study, Provencher et al. showed that open RI repair had more external rotation loss than arthroscopic closure because the contribution and strain of the coracohumeral ligament (CHL) and SGHL were more pronounced in the neutral rotation and exhibited greater rotation restraint [9]. However, in the cadaveric study, open RI repair and arthroscopic RI closure were performed with the glenohumeral joint in the 30° of external rotation and neutral abduction position. It might be important to place the shoulder in some degree of external rotation and abduction to avoid over tightening and potential postoperative loss of external rotation. Nobuhara and Ikeda showed that open RI repair was performed with the shoulder in an 90° externally rotated position, ensuring a close fit of the SSP and SSC attachments [2]. In this previous study, 91% of patients had a full range of motion postoperatively.

There are several arm positions reported for arthroscopy RI closure. Arthroscopic RI closure has been described in the 30° of abduction and external rotation [10], 45° of abduction and external rotation [11], and 90° of external rotation positions [12]. RI closures have been described for lesions as well as an adjunct to instability surgery. Surgeons often perform a RI closure to purposely limit external rotation and to help limit humeral head translations.

However, in our case, we observed the shoulder joint by arthroscopy after open RI repair. The point of RI repair was not observed when there was >30° of external rotation. This might indicate that RI closure under arthroscopy might be difficult to perform in the 90° externally rotated and abducted position, which would ensure a close fit of the SSP and SSC attachments. Wang et al. showed that increasing the maximum external rotation of the shoulder would increase the ball velocity because of higher linear and angular displacement of the throwing forearm [13]. To prevent postoperative external rotation loss of the shoulder and reduction in performance for a baseball pitcher, we recommend that RI repairs and closures be performed with the shoulder in the 90° externally rotated and abducted position.

In conclusion, open RI repair in the 90° external rotated and abducted position leads to good clinical results without postoperative loss of external rotation. This method might be considered an effective option for treating RI lesions for patients requiring high performance, such as baseball pitchers or throwing athletes.
